# An Interesting Case of Bilateral Facial Palsy due to Granulomatosis with Polyangiitis

**DOI:** 10.1155/2021/9963564

**Published:** 2021-08-04

**Authors:** Rajish Sanjit Kumar Shil, Jamal Ali Teir

**Affiliations:** ^1^Department of Internal Medicine, Medical Institute, Al Ain Hospital, Al Ain, UAE; ^2^Department of Rheumatology, Medical Institute, Al Ain Hospital, Sheikh Khalifa Hospital, Abu Dhabi, UAE

## Abstract

Granulomatosis with polyangiitis (formerly called Wegener's granulomatosis) is a systemic autoimmune disease, which can lead to necrotizing vasculitis affecting small vessels and cause inflammation of blood vessels in the nose, sinuses, throat, lungs, and kidneys. In rare instances, it has shown involvement of the brain and cranial nerves as well. We are reporting a case of granulomatosis with polyangiitis, complicated by bilateral facial palsy due to lower motor neuron involvement of the facial nerve, which has responded well to immunosuppressive treatment, particularly rituximab. It is prudent to be vigilant in investigating patients with atypical presentation for systemic autoimmune diseases, as this approach would affect the patient morbidity and mortality with early initiation of treatment for the disease.

## 1. Introduction

Granulomatosis with polyangiitis (GPA) is a necrotizing vasculitis affecting both arterioles and venules. The disease classically presents with a triad involving acute inflammation of the upper airways and lower airways along with renal involvement. However, it is known that GPA can affect any organ system. Cranial involvement can result in cranial nerve palsy and, rarely, pituitary infiltration.

## 2. Case Description

A 47-year-old female, previously known to have type 2 diabetes mellitus, was admitted to a tertiary-care hospital in Al Ain city, with complains of severe headache, dizziness, and right-sided facial weakness. She has been complaining of headache and dizziness for a duration of 2 months, which has been ongoing intermittently, but has increased in the past 5 days prior to admission, associated with right ear pain. She described headache as of pulsatile in quality, more on the right side, with a severity of 7/10, increasing with bending forward, relieved with low light condition and analgesics. Her dizziness was in the form of vertigo, increased with head movements, more on the right side. She had right lower motor neuron (LMN) type facial palsy, which developed around two months prior to current presentation and has been improving with a course of steroid treatment. In addition, two weeks prior to her development of right lower motor neuron facial palsy, she had a history of left lower motor neuron facial palsy, which was treated with a course of systemic steroids. She has a long-term history of chronic maxillary sinusitis and bilateral otitis media, complicated with conductive hearing loss, which was treated with bilateral ear grommet tube insertion, three months prior and is on regular follow-up with the ENT team in a private hospital. She had no other symptoms or signs of vasculitis.

**Figure 1:**
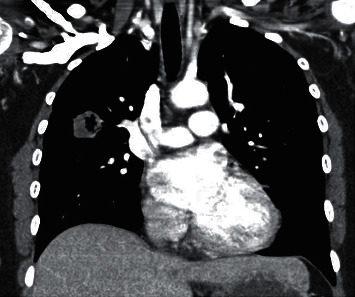
CT thorax showing the cavitary lesion in the right upper lobe of the lung.

**Figure 2:**
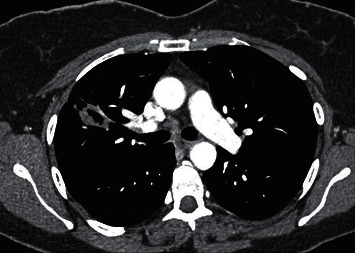
CT thorax axial view showing the cavitary lesion in the right upper lobe of the lung.

**Figure 3:**
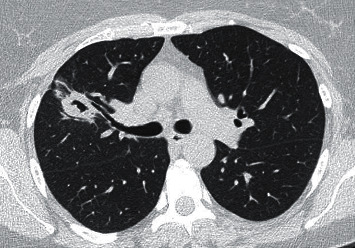
CT thorax (lung window) axial view showing the cavitary lesion in the right upper lobe of the lung.

**Figure 4:**
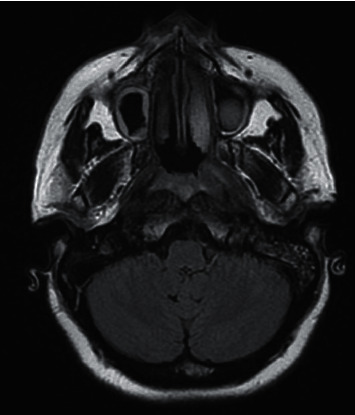
MRI brain axial view showing bilateral maxillary sinusitis and left mastoiditis.

Along with recurrent history of otitis media and sinusitis, she also had history of fever and cough, which was treated as for pneumonia in another hospital facility, following which, on admission to our hospital, her chest X-ray revealed a patchy opacity in the right mid zone, which was further investigated via computed tomography (CT) thorax (Figures 1–3) and biopsy. CT thorax revealed a cavitary lesion in the right upper lobe measuring about 2.5 × 2.5 × 1.9 cm, which has thick irregular outline with nearby interstitial changes. Magnetic resonance imaging (MRI) of the brain revealed significant sinusitis of both the maxillary sinuses and mastoid air cells of both sides, with normal findings of the brain (Figure 4).

Further investigation including autoimmune screening showed that she had raised erythrocyte sedimentation rate (ESR) level (62 mm/hr), raised C-reactive protein (CRP) levels (54 mg/L), positive C-antineutrophil cytoplasmic antibody (ANCA) level (106RU/mL), mild lymphopenia (1.15 × 10^∧^9/L), mild iron deficiency anemia (hemoglobin 95 g/L), elevated lactate dehydrogenase (LDH) level (322 IU/L), and raised HbA1c 6.8%. Other parameters for urea, creatinine, electrolytes, and urine tests were normal, and there was no evidence suggestive of any form of renal involvement. She tested negative for blood and urine cultures.

Workup was negative for *p* ANCA, antinuclear antibody (ANA), rheumatoid factor (RF), cyclic citrullinated peptide (CCP), and quantiferon-TB, and she also had normal angiotensin-converting enzyme (ACE) levels (25 microliters). Her HIV and hepatitis screen was also negative. The patient was advised to undergo lumbar puncture for further investigation for headache, to which she refused. Facial nerve palsy was diagnosed clinically, and no additional tests were performed to confirm the diagnosis at this instance.

The histopathological report from the CT-guided biopsy of the lung lesion revealed findings of extensive coagulative necrosis and moderate interstitial inflammation comprising lymphocytes, neutrophils, histiocytes, plasma cells, and eosinophils. There were also scattered multinucleated giant cells of langhans type and vague granulomatous and focal neutrophilic infiltration consistent with leucocytic vasculitis. It was negative for acid-fast bacillus and fungal strains. The histological features were compatible with the diagnosis of granulomatosis with polyangiitis.

Given the histopathological findings and positive C-ANCA levels, a diagnosis of granulomatosis with polyangiitis was made and it was decided to start treatment with immunosuppressants. The involvement of the cranial nerves causing bilateral facial palsy is presumed to be due to the autoimmune inflammatory process from granulomatosis with polyangiitis, as it is unusual to have bilateral Bell's palsy apart from diseases such as sarcoidosis, Lyme disease, Guillain–Barre syndrome, meningitis (neoplastic or infectious), and rarely congenital.

The patient was started on treatment with analgesics for headaches and beta-histine for peripheral vertigo as per the ENT team recommendations initially. After multidisciplinary team discussion, the patient was started on intravenous (IV) pulse steroid with methylprednisolone 1 gram/day for 3 days, followed by oral prednisolone 60 mg per day. Following completion of pulse steroid treatment, she was started on IV rituximab 1 gram as per rheumatology recommendations and also carefully taking into account the patient's denial to receive IV cyclophosphamide due to fear of the side effects and renal impairment in the long run. This approach was supported by the fact that she had normal renal function and as per the literature, and both immunosuppressants showed equal efficacy in treatment for GPA. The patient had significant improvement after starting the treatment, and she was followed up regularly in the rheumatology clinics.

Prior to starting of the immunosuppressants, the calculated Birmingham Vasculitis Activity Score/Wegener's granulomatosis (BVAS/WG) was 27. She received her second dose of rituximab 1 gram after 2 weeks, following the first dose, and her symptoms showed significant improvement. After 3 weeks, her BVAS score came down to 15 and she had almost 90% improvement in her headache, dizziness, facial palsy, and numbness. Her thiopurine methyltransferase (TPMT) enzyme level activity was slightly low towards the borderline, but not deficient and had a normal genotype. Considering this, she was started on a slightly lower dose of azathioprine 50 mg daily, later increased to 100 mg daily and was kept on close monitoring. Steroid treatment was continued on a tapering dose, and she was also kept on bone protection agents such as alendronate, vitamin D, and calcium supplements. She was kept on prophylaxis treatment for pneumocystis jeroveci infection with co-trimoxazole and also supplemented with ferrous fumarate for anemia.

Following two months of treatment with regular follow-up, her inflammatory marker had improved to normal with C-ANCA level 7 RU/mL, CRP level 6.4 mg/L, and ESR 10 mm/hr. Her complete blood count showed normal hemoglobin, leukocytes, lymphocytes, neutrophils, and platelets. Steroid treatment was tapered slowly. She was kept on azathioprine 100 mg per day, calcium, vitamin D, and co-trimoxazole along with her diabetic and hypertension medications. She continued to be followed up regularly in the ENT, diabetes, and rheumatology clinic to be monitored for any further complications.

## 3. Discussion

GPA is a systemic inflammatory disease which presents with necrotizing granulomas and vasculitis in multiple organs. The prevalence of GPA is around 3 per 100,000 population [1]. It usually presents with head, neck, pulmonary, and renal manifestations [2]. Nasal and paranasal sinus disorder is observed in 90% of the cases and are the most common primary manifestations. The second most common manifestation in GPA is usually pulmonary infiltrates or nodules. Ear disorders can be seen in 20%–61% of patients, but are rarely the first and only manifestation [3]. Nervous system involvement is also rarely reported at the initial stage, but mononeuropathy multiplex can develop in 15% and facial palsy in 5% of the patients [2]. Bilateral facial palsy is an unusual manifestation, and its precise incidence is unclear. Reviewing the current literature, a study from the Mayo Clinic found that almost 30% of 324 patients had neurological involvement [4]. The cranial nerves most commonly affected were the optic nerve, abducens nerve, and facial nerve, while in some cases, the glossopharyngeal, vagus, and hypoglossal nerves were less commonly affected. Notwithstanding the common neurological involvement, cranial neuropathy as the first manifestation of the disease preceding other organ involvement is uncommon.

Our patient had bilateral facial nerve involvement, but both occurred between the time spans of two weeks. The patient was suffering from bilateral otitis media with effusion for a prolonged period, which required Grommet tubes to be inserted. There is a possibility that the facial nerve involvement could be due to the disease process in the middle ear due to segmental nerve compression on its course in the auditory canal, which led to the start of the facial palsy on one side, and later, it manifested on the other side. Other possibility is that it was the vasculitis disease process affecting the blood vessels of the seventh cranial nerve, which led to the palsy. Furthermore, the interval between facial palsy was not very long as seen in our patient as she was at the recovery stage of her left-sided LMN facial palsy with treatment with steroids, when she developed the right-sided LMN facial palsy. Since the patient has shown significant improvement with the steroid and immunosuppressant treatment, we can confidently imply that the disease process was related to vasculitis rather than the middle ear disease process contributing to the facial palsy. A comparison of similar cases reported in the literature has been depicted in Table 1.

The combination of bilateral advanced hearing loss and unilateral facial palsy as presenting signs of the disease is very uncommon [5]. Occasionally, due to the progressive nature of the disease process, it may lead to bilateral facial palsy, which was clearly observed in our patient. Bilateral facial palsy is an extremely rare finding [6]. In a review of 2856 patients with facial paralysis, Lee et al. reported only 2% of cases with bilateral involvement [7]. During GPA with bilateral ears disorders, bilateral facial palsy develops extremely rarely with only few cases reported in the literature [7].

Our patient was diagnosed based on the clinical presentation of upper airway involvement, i.e, chronic sinusitis and otitis media, positive C-ANCA level, and histopathological findings of GPA from the lung lesions. She did not have any evidence of renal involvement at the time of diagnosis, and this was taken into consideration for her treatment with rituximab rather than cyclophosphamide, along with the fact that the patient denied receiving IV cyclophosphamide, and as per the literature, both immunosuppressants showed equal efficacy [8]. The patient responded very well to this treatment regimen, as she is being followed up regularly in the rheumatology clinic till date.

Prognosis of neuropathies associated with Wegener's granulomatosis depends mainly on early diagnosis and prompt initiation of treatment [9–11].

## 4. Conclusions

Granulomatosis with polyangiitis is an autoimmune disease, rarely affecting the cranial nerves, and the disease can be lethal, if left untreated. Long-term remission can be achieved in up to 90% of the cases with the help of therapeutic agents such as steroids and other immunosuppressant drugs such as cyclophosphamide, rituximab, azathioprine, and methotrexate. Early detection of the disease process and initiation of treatment will improve the patient outcomes and minimize the complications of the disease to occur in such patients.

## Figures and Tables

**Table 1:** Comparison of similar reported cases in the literature.

Authorship	Report	Presentation	Treatment
A. R. et al., 2013	Bilateral facial palsy in rapidly progressive course of Wegener's granulomatosis: a case report	Initially presented with bilateral otitis media, which progressed to bilateral facial palsy	Antromastoidectomy, decompression, and methylprednisolone followed by cyclophosphamide

A. W. et al., 2016	Granulomatosis with polyangiitis with bilateral facial palsy and severe mixed hearing loss	Presented with bilateral otitis media, left facial palsy, and later progressed to bilateral facial palsy	Myringoplasty, antromastoidectomy with facial nerve decompression, antibiotics, methylprednisolone, and cyclophosphamide

R. U. et al., 2016	A case of Wegener's granulomatosis presenting with unilateral facial nerve palsy	Presented with left-sided facial palsy, with preceding ongoing persistent rhinorrhea	Methylprednisolone, cyclophosphamide, antituberculosis treatment, low-dose steroid, and azathioprine

P. D. et al., 1998	Otologic Wegener's granulomatosis with facial nerve palsy	Presented with acute otomastoiditis with facial nerve involvement	Urgent mastoidectomy with facial nerve decompression

R. S. et al., 2021 (present case)	Bilateral facial palsy due to granulomatosis with polyangiitis	Presented with chronic maxillary sinusitis, bilateral otitis media, vertigo, and left-sided facial palsy, followed by right-sided facial palsy	Oral steroids, bilateral Grommet tube insertion, methylprednisolone, rituximab, and azathioprine

## Data Availability

The radiological imaging and laboratory data were collected from electronic medical records and were used to support the findings of this study for the publication of this individual case report.

## Abbreviations

GPA: Granulomatosis with polyangiitis
LMN: Lower motor neuron
CT: Computed tomography
MRI: Magnetic resonance imaging
ESR: Erythrocyte sedimentation rate
CRP: C-reactive protein
LDH: Lactate dehydrogenase
ANA: Antinuclear antibodies
CCP: Cyclic citrulinated peptide
RF: Rheumatoid factor
ACE: Angiotensin-converting enzyme
ENT: Ear, nose, throat
IV: Intravenous.
